# Pigmented Potatoes: A Potential Panacea for Food and Nutrition Security and Health?

**DOI:** 10.3390/foods11020175

**Published:** 2022-01-10

**Authors:** Callistus Bvenura, Hildegard Witbooi, Learnmore Kambizi

**Affiliations:** Department of Horticulture, Faculty of Applied Sciences, Cape Peninsula University of Technology, Bellville 7535, South Africa; Callistusb@gmail.com (C.B.); hildegardwitbooi@gmail.com (H.W.)

**Keywords:** anthocyanins, carotenoids, food and nutrition security, pigmented potatoes, polyphenols, sustainability

## Abstract

Although there are over 4000 potato cultivars in the world, only a few have been commercialized due to their marketability and shelf-life. Most noncommercialized cultivars are pigmented and found in remote regions of the world. White-fleshed potatoes are well known for their energy-enhancing complex carbohydrates; however, pigmented cultivars are potentially high in health-promoting polyphenolic compounds. Therefore, we reveal the comprehensive compositions of pigmented cultivars and associated potential health benefits, including their potential role in ameliorating hunger, food, and nutrition insecurity, and their prospects. The underutilization of such resources is a direct threat to plant-biodiversity and local traditions and cultures.

## 1. Introduction

The COP26 Glasgow meeting could not have come at a better time, when extreme weather events such as floods, uncontrollable fires resulting from heat waves, heavy downpours, droughts, hurricanes, and heavy snow have become the new norm the world over. In fact, the warmest temperatures, the highest sea level rise as well as warming, and the consequent sea acidification over the past seven years have been the highest on record [1]. Perhaps one of the major concerns about global warming is its negative impact on livelihoods and, consequently, food and nutrition security. The COVID-19 pandemic also came with devastating effects on the state of food security in the world due to strict lockdown regulations. Reports indicate that world hunger increased in 2020 due to the pandemic. More specifically, in the year 2020, undernourishment increased by 1.5%, with 218 and 418 million of these cases residing in Africa and Asia, respectively [2]. More than a third of the world’s population did not have access to adequate food in 2020 and 12% were severely food-insecure. Reports further indicate that due to financial and other factors, healthy diets were out of reach for 3 billion people in the world in 2020 [2]. Unhealthy diets cost governments billions of USD in health care annually. Therefore, climate heating, extreme weather events leading to the loss of livelihoods and droughts, and increasing global populations, among other factors, are making it nearly impossible to achieve the UN SDGs to feed the world by 2030.

Malnutrition resulting from unhealthy diets, food insecurity, and other factors have also been on the rise, with statistics indicating that in the year 2020 alone, overweight, wasting, and wasting affected 5.7%, 6.7%, and 22% of children under 5 years, respectively [2]. Most of these cases emanate from Africa and Asia. Malnutrition resulting from diets that do not supply a healthy amount of nutrients has become a major problem in the world today. These diets are normally ladened with highly processed foods that are high in free sugars, saturated fats, and salt and are very low in the recommended daily intakes of fruits and vegetables. Unhealthy diets are the major cause of noncommunicable diseases (NCDs). NCDs, which were associated for a long time with more developed and industrialized countries of the world, have become epidemics the world over, including in those of the poor. Over 70% of global mortalities have been linked to NCDs, and over 77% of these figures have been reported in low- to middle-income countries [3]. Diabetes (1.5 million), respiratory diseases (4.1 million), cancer (9.3 million), and cardiovascular diseases (17.9 million) dominate the global NCD deaths [3]. Although some metabolic risk factors (such as hypertension, obesity, and glycemia) and lifestyle behavior (including smoking, lack of physical activity, and stress) do contribute to NCD, it must be noted that unhealthy unbalanced diets remain the major cause. The WHO and many other organizations are teaming up to encourage the consumption of healthy diets and making use of some indigenous underutilized foods. Most of these foods have been proven to be healthy and have the potential to ameliorate the current unhealthy societies that have been bred over the past few decades. Staple food crops (potatoes, maize/corn, wheat, rice, cassava, sweet potatoes, sorghum, and yams) are an important aspect of the diet because they supply the much-needed carbohydrates for energy. However, these need to be nutritionally balanced and in the right quantities to breed healthy nations. To fully appreciate and increase the pace toward a food- and nutrition-secure world, these crops need to be understood and studied at length. Therefore, in this review, we pay attention to the potato, with special focus on the little-known and underutilized pigmented cultivars.

**Figure 1:**
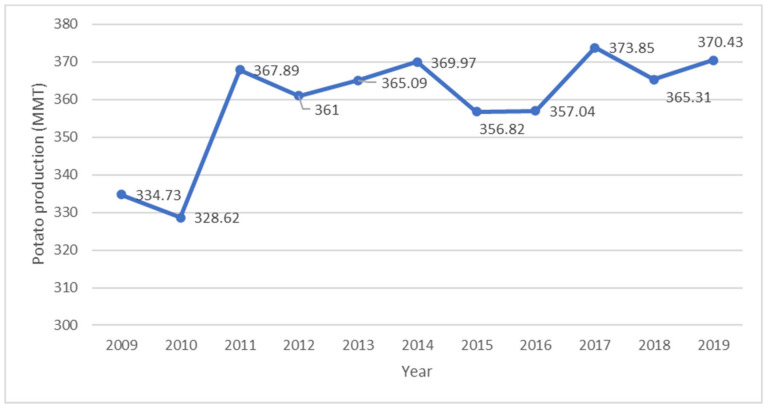
World potato production from 2009 to 2019 [4].

**Figure 2:**
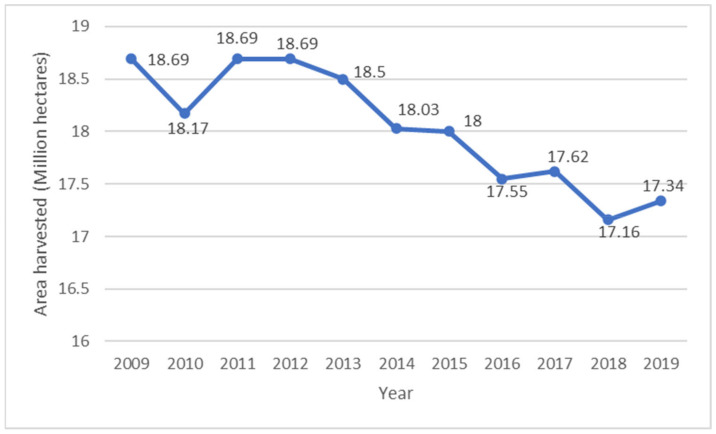
Total area under potato production in the world from 2009 to 2019 [6].

In general, the potato (*Solanum tuberosum* L.) is one of the most important food crops that feed the world, after rice and wheat, with over a billion people being sustained by the crop and production now exceeding 350 MMT annually harvested on 17 million hectares of land, as shown in Figure 1 [4]. In the past ten years, statistics indicate that production has increased by 11%. However, this is a decline in comparison with a 21% increase that was reported by [5] between 1991 and 2007. Although there has been an increase in potato production in the world, a 7% decline in total harvested area between 2002 and 2019 has been reported, as shown in Figure 2 [6]. Up-to-date statistics also indicate that the world per capita consumption of potatoes was 32.3 kg in 2018, and this was a decline by 3.76% from 2017 and 2.22% decline from 2008 [7]. Belarus (182 kg), Ukraine, Rwanda, Latvia, Kazakhstan, Russia, Poland, Romania, Kyrgyzstan, and Peru are the top ten highest-potato-consuming countries of the world per capita [7].

**Figure 3:**
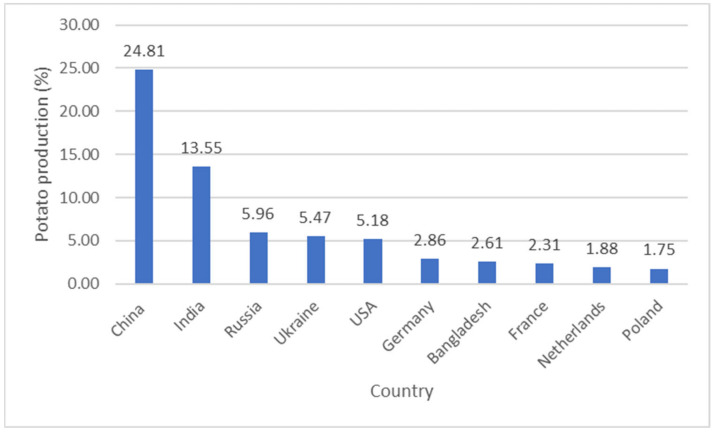
World’s leading potato producers in 2019 [9].

In addition to being the largest vegetative-propagated crop worldwide, the potato tuber has become an important staple in parts of the world where there is a limited but increasing purchasing power, an increasing pressure on scarce land, and an increasing demand for food [8]. This is because the potato is a short season crop and is often replanted as a seed potato, in addition to requiring minimum inputs [4]. First used as food and domesticated from the wild in the Andes region of South America over 8000 years ago, the potato has adapted very well to all regions of the world, except in Antarctica where the soil and conditions are untenable for crop production [5]. The crop was first introduced to Europe in the 1570s after which it spread to the rest of the world in the late 17th century [5]. As shown in Figure 3, China, India, and Russia are the current top three potato producing countries in the world [9].

Although over 4000 potato cultivars are known today, only a few have been successfully commercialized because of their shelf-life stability and marketability, and none of these are pigmented [10]. For producers and sellers of potatoes, the ability to be stored for long periods of time, a regular-sized tuber, multipurpose use, high production ratio, and customer acceptance are some of the critical characteristics to consider to turn a profit and avoid wasting of the product [11]. Common potato cultivars are white-fleshed, while the pigmented cultivars are rare. According to the same authors, potato farmers in the world are reluctant to cultivate these pigmented potatoes due to their low yielding capacity, as well as their susceptibility to diseases during seed multiplication. Therefore, in this review, we explore pigmented potato cultivars and their possible role in food and nutrition security. We bring to the fore the phytochemical characteristics that have been reported, their biological activities, and how these can be harnessed into breeding healthier nations of the world.

## 2. Pigmented Potato Cultivars

**Figure 4:**
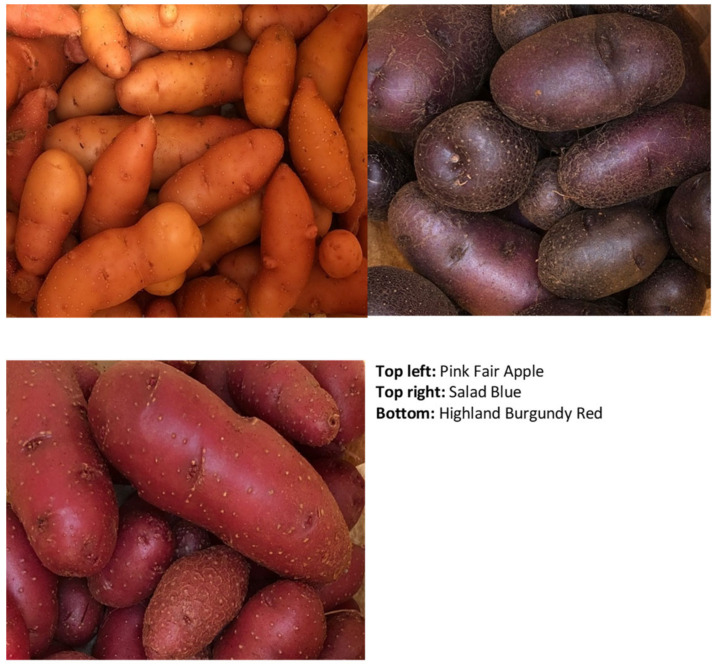
Some pigmented cultivars from South Africa.

The literature does not give a specific total number of pigmented cultivars, although a general number exceeding 4000 for all potatoes, including the white-fleshed cultivars, has been reported in the world [12], and, according to [13], nearly 1000 Andean species, many of which are pigmented. An exact number of pigmented species will most likely remain elusive because most of these species are cultivated in remote regions of the world for subsistence purposes and will remain hidden and/or be eventually driven to extinction if nothing is done to preserve them. Variations of red, purple, blue, and yellow skin and/or flesh have been reported in the literature as the dominant colors across the world (Table 1). Pictorial examples of some pigmented cultivars from South Africa are shown in Figure 4. These colors are mainly due to the presence of anthocyanins and other pigments. Although it is assumed that at least 50% of the potato tuber is consumed fresh, this number is conceivably higher for pigmented potatoes [5]. This is because the white-fleshed cultivars can also be developed into products and ingredients, animal feed, and industrial applications such as starch. The white-fleshed cultivars are produced in millions of tons annually and, therefore, multiple uses can be generated. Pigmented cultivars are produced on a small scale and, therefore, food applications dominate their uses. In fact, the internet is awash with gourmet applications of the pigmented cultivars in high-end restaurants. Food lovers are perhaps attracted by their unique and beautiful colors and, least of all, by their nutritional and health benefits. Ref. [14] worked on extracts of red and purple cultivars as potential food colorants. Such studies would bring to the fore the potential of these cultivars and help to improve on their size, shelf life, and their marketability. Several genetic studies have been undertaken on these species, including those of [15,16]. More research needs to be channeled toward the development of better cultivars.

## 3. Antioxidant Activity in Pigmented Potatoes

**Figure 5:**
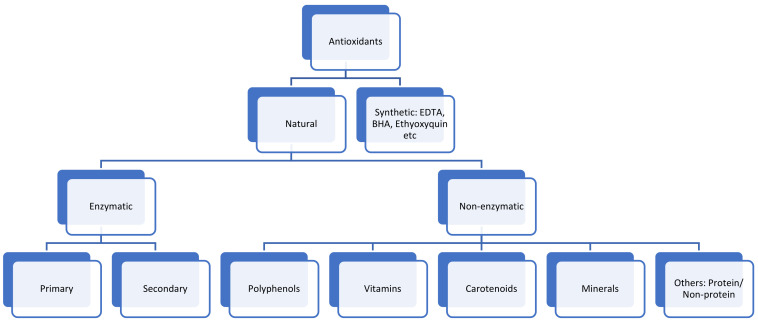
Schematic representation of antioxidants.

Over the last decade, there has been a growing interest and preference of consumers for food containing natural antioxidants for health and nutritional-related reasons. In any case, food regulatory institutions demand that food products should contain labels that specify the ingredients but should not include any health claims. However, whether the consumers understand what antioxidants are remains an open question. In human physiology, normal cellar metabolism produces reactive oxygen species (ROS), which play a positive role in physiological processes but only in low concentrations [74]. In high concentrations, these ROS become toxic and cause damage to proteins, DNA, and lipids [75]. Therefore, antioxidants stop the damage caused by these ROS. Antioxidants are therefore a good indicator of the health benefits that can be derived from the foods. A simple schematic presentation of antioxidants is given in Figure 5, showing an overview of these compounds.

The rich antioxidant potential of pigmented potatoes in comparison with white-fleshed cultivars has been scarcely reported in the literature. In fact, the study of [42] showed a reduction in chronic disease susceptibility, inflammation, and cellular oxidative damage in adult males. These authors’ study showed the antioxidant potential of pigmented potatoes. Furthermore, the in vivo study of [35] revealed the high antioxidant activity of purple potato flakes through RNA and linoleic acid oxidation inhibition. These authors further showed that hepatic Cu/Zn-SOD, GSH-Px mRNA, and Mn-SOD expression is further improved by the purple potato flakes’ antioxidant activity. More studies have shown that pigmented potatoes possess high antioxidant activity and possess the potential to reduce oxidative stress [17,18,19,25,35,48,49,53,66,73]. Therefore, the consumption of pigmented potato cultivars could potentially offer better health benefits in comparison with the traditional white-fleshed cultivars. Health benefits associated with antioxidants such as anticancer, antiaging, and anti-inflammatory activities could potentially be derived from the consumption of these species.

## 4. Carotenoids

**Figure 6:**
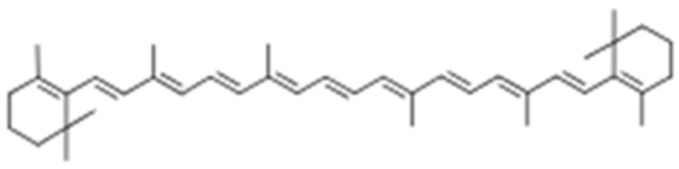
General structure of carotenoids—β-carotene [78].

Several studies have reported on carotenoids in pigmented potato cultivars. This is because carotenoids are the basic source of orange, yellow, and red pigments in plants and are also widely distributed in nature, including in algae, bacteria, and photo as well as non-photosynthetic organism tissues [76]. The basic structure of a carotenoid is shown in Figure 6. Although α and β carotenes are known for provitamin A activity, they cannot be synthesized by the body and therefore needs to be sourced from food. Carotenoids are very common in vegetables and fruits such as peaches, spinach, apricots, carrots, butternut, and sweet potatoes. Although over 700 carotenoids are known to exist in nature, about 24 are common in the food that we consume and only four have been exhaustively studied, viz., α-carotene, β-carotene, lycopene, lutein, and zeaxanthin [77].

Based on their chemical structures, the role of dietary carotenoids in quenching harmful reactive oxygen species (ROS) and intercepting toxic free radicals is well reported [79]. Carotenoids are therefore important natural antioxidants that form a strong defense against harmful biological processes. The defensive role of carotenoids against muscular degeneration, cardiovascular diseases, and a wide range of cancers including uterine, lung, colorectal, prostate, and breast have been reported [80]. Reports also reveal the protective effects of lycopene, β-carotene, and lutein against the formation of erythema, which is caused by exposure to UV light [81]. Therefore, it is not surprising that carotenoids play a significant role in the cosmetics industry, which is worth billions of USD in the world today [82]. Some of the carotenoids that the pharmaceutical industry has been able to synthesize in the laboratory for the cosmetic industry include astaxanthin, β-carotene, canthaxanthin, lycopene, and zeaxanthin [76].

Nevertheless, the current literature search revealed several studies that have documented carotenoids in pigmented potato cultivars. The study of [26] found that yellow-pigmented cultivars possessed between 280% and 2000% more carotenoids than the white-fleshed cultivars depending on pigment intensity. The intensely yellow cultivars possessed more carotenoids, while lutein and xanthophyll were the dominant compounds. Some authors profiled 60 pigmented potatoes and found that the major carotenoids to dominate the cultivars were neoxanthin, lutein, and violaxanthin in 7, 16, and 37 cultivars, respectively, and the total compositions ranged between 50.0 and 1552.0 µg/100 g [29]. Minor carotenoids such as zeaxanthin, antheraxanthin, β-carotene, and β-cryptoxanthin were also reported by the previous authors. In another study, Ref. [52] found that total polyphenols and carotenoids increased in purple potatoes in response to conventional cultivation as opposed to organic cultivation. These authors reported a positive correlation between the color of the potatoes and polyphenol and carotenoid contents, although the carotenoids ranged between 0.012 and 0.085 mg/100 g. The two-year study of [36] in the Czech Republic found that genotype, soil type, and year of growth in pigmented cultivars affected the carotenoid contents and ranged between 0.779 and 13.3 mg/kg. Lutein dominated the carotenoids in their study, while β-carotene, zeaxanthin, neoxanthin, and violaxanthin were also found but in marginal proportions in all the cultivars. Additionally, the yellow cultivar Agria possessed higher levels than the blue and red types. To corroborate this study, [67] found that location, genotype, period of growth, and their interactions in Poland significantly affected carotenoid contents in some pigmented cultivars grown in four different locations for three consecutive years. The contents were higher than in the Czech Republic and ranged between 5.57 and 20.20 mg/kg, and lutein dominated (2.92 to 6.66 mg/kg) more than zeaxanthin (1.44 to 3.05 mg/kg). The Belgian study of [18] revealed that zeaxanthin and lutein contents in 23 pigmented cultivars ranged between 0 and 17.7 µg/g and between 1.12 and 17.69 µg/g, respectively, while β-carotene ranged between 0.42 and 2.19 µg/g in 16 cultivars. β-carotene is rarely reported in potato tubers, perhaps a strong indication of the elevated carotenoid contents in these species. The study of [70] revealed that the carotenoids lutein and β-carotene were more elevated in potatoes cultivated under a biodynamic system, in comparison to those that were organically produced. These studies indicate that carotenoids in pigmented potatoes are cultivar-, pigment-, intensity-, and environment-dependent. The yellow-pigmented cultivars are generally higher in carotenoids in comparison to other cultivars, and more so, the white-fleshed type and lutein appear to dominate these compounds. In human physiology, lutein is found concentrated in the macula of the retina in the eyes. Although the precise lutein role is not yet fully known and understood, its association with vision cannot be farfetched. Therefore, the consumption of these carotenoid-rich potatoes could potentially act as a buffer against the threat of lifestyle and diet-induced diseases and conditions and help in the management and treatment of vision-related issues.

## 5. Anthocyanins in Pigmented Cultivars

**Figure 7:**
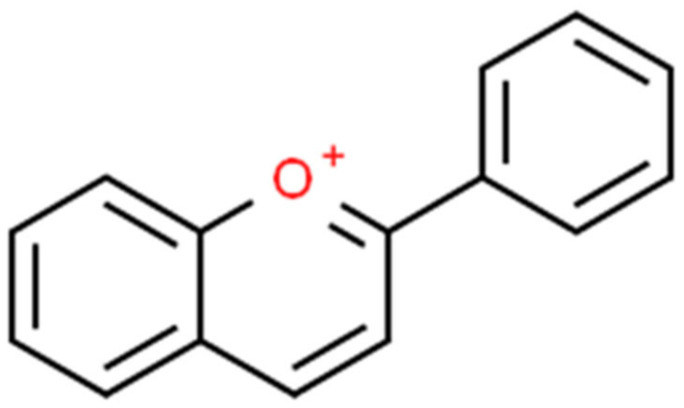
Basic anthocyanin structure—flavylium [86].

Most phytochemical studies that have been conducted on pigmented cultivars are on anthocyanins. This is because of the color of these species’ skin and/or flesh. Anthocyanins are water-soluble phenolic compounds that fall under the flavonoid subgroup [83]. Anthocyanins occur in plants as glycosides attached to a sugar group, as shown in Figure 7. Located in the vacuole, these compounds give plants their distinctive color and are widely distributed in vegetables and fruits, such as strawberries, blueberries, blackberries, currents, mulberries, blackcurrant, and red/blue grapes [84]. The purple to blue and red pigments given off by the action of anthocyanins function to attract animals to consume the fruits and aid in seed dispersal, while in flowers, pollinators are also attracted [83]. In addition, the bright colors help to absorb radioactive ultraviolet and blue-green light and act as a plant sunscreen. Peonidin, petunidin, cyanidin, malvidin, delphinidin, and pelargonidin are six widely distributed anthocyanins from a total of 17 that are known to be found in nature [84]. However, [85] had earlier argued that there are nearly a thousand anthocyanins in Kingdom Plantae. Nevertheless, when the pH is acidic, anthocyanins are thought to exhibit a positive charge in their structure [84]. Furthermore, at pH < 2, anthocyanins are said to exist as flavylium, a basic and stable compound. Consequently, anthocyanin’s bioavailability, metabolism, absorption, and biological responses are thought to be influenced by these unique chemical structures [84]. According to the previous authors, neutral and alkaline pH have a degrading effect on anthocyanins, with a bioavailability as low as 0.1%. Understanding the behavior of this antioxidant is critical in understanding its role in health and disease management, especially NCDs, which are often negatively affected by poor and unhealthy diets. Several studies have documented the anthocyanin antidiabetic, antiproliferative, and antioxidant, as well as improved insulin resistance in nonalcoholic fatty liver disease (NAFLD), risk factor reduction in cardiovascular diseases and an improvement in eyesight among other benefits [28,85,86].

As shown in Table 2, the current literature search was able to reveal petunidin, cyanidin, delphinidin, petunidin, pelargonidin, peonidin, and malvidin that have been profiled and quantified in these species. The study of [73] showed that anthocyanins were more elevated in the skin of pigmented potatoes in comparison to the flesh. Jansen and Flamme [39] also found the skins to contain more anthocyanins than the flesh, while the whole tuber compositions lied between the skin and the flesh. The previous results have also been corroborated by [71]. Therefore, the current literature search is a good indicator of the potential of pigmented potato cultivars as health-bearing underutilized foods. Anthocyanins are, in fact, the highest-consumed flavonoids with an estimated intake of 200 mg/d, therefore making them valuable components of the diet [85]. The use of the skin for both food and industrial applications or color dyes should therefore not be ignored, as anthocyanins are more elevated in these organs.

## 6. Pigmented Potato Biological Activity

The antioxidant activity and anthocyanins contained in pigmented potatoes are a good indicator of some potential biological activity. Few studies have been conducted to determine the biological activities of these species; however, a few that have been conducted open insights into endless possibilities on the applications of these species in diverse areas of life. Anticancer studies of pigmented potato cultivars are scarce but look promising. Although the in vitro study of [72] showed that the ethanolic extracts were minimally active against the hepatocellular carcinoma cell lines, these need to be tested against other cell lines to confirm these findings. The study of [68] showed a reduction in cancer incidence and multiplicity, but in vivo, when they used the extracts of pigmented potatoes. The study of [20] also revealed that the extracts of some pigmented potatoes were lethal against the hepatocarcinoma (Hep3B) cell lines. Silveyra et al. [65] also showed that the skin and flesh extracts of some cultivars possessed some antibacterial properties against *E. coli*. These authors reported that the skin possessed more activities than the flesh extracts. These studies are promising and should further be addressed toward other yet-to-be-researched cell lines. Pigmented potatoes could potentially hold the key to solving the seemingly never-ending cancer plague that the world is facing today. If polyphenols are profiled, some of the compounds could be useful as anticancer compounds. Unfortunately, exploratory studies to exhaustively profile polyphenols in these species are lacking. This knowledge gap needs to be filled.

## 7. The Impact of Environmental, Genotypic, and Soil Types on Pigmented Potato Characteristics

The effects of environmental factors on the growth of plants have been widely studied and reported. A few studies of this nature have been conducted on pigmented cultivars, and the results offer some insights into commercialization prospects of these species. Witbooi et al. [11] subjected pigmented potatoes to various root zone temperatures and found that 24 °C significantly increased plant height and tuber weight in all cultivars, while 28 °C increased polyphenols. Gutiérrez-Quequezana et al. [33] reported that 13 and 18 °C did not affect either anthocyanins or polyphenolics; however, the increase in anthocyanins at maturity in purple cultivars and high polyphenolics in the blue cultivars were highly cultivar-dependent. In another study, Ref. [39] showed that nitrogen fertilizer did not affect pigmentation in potatoes. Meanwhile, Ref. [26] reported that the growing environment, including altitude and soil factors, significantly increase anthocyanin production but have no effect on carotenoids. A Korean study [41] showed that anthocyanins responded well to high-altitude areas in comparison to those cultivated in a low-lying area over a two-year period and in 14 different locations. These authors also reported a negative correlation between soil acidity and anthocyanin contents. However, in Poland, the effect of environmental factors, genotypes, the year, and interaction of these factors had a significant effect on carotenoid contents [67]. Andre et al. [19] showed that the effect of drought stress on potato phytochemicals was highly cultivar-dependent and varied between cultivars. However, antioxidant contents were weakly affected by drought stress in yellow cultivars, while polyphenols and anthocyanins were drastically reduced in purple and red cultivar flesh. The study of [70] showed that organically and biodynamically produced cultivars had higher anthocyanin and polyphenolic compounds, although biodynamic cultivars had significantly higher carotenoids in comparison with the control. In a similar study, [52] found that the conventional cultivation of pigmented cultivars increased total polyphenols and carotenoids in comparison to those that were organically produced. In Texas and Colorado USA, anthocyanins and polyphenols decreased in the tubers as they matured, but the yield and compounds increased [58]. Harvesting time is thus useful if the maximum compound yield is the objective. Some breeding studies have also been conducted on high-anthocyanin-yielding clones to understand their breeding behavior. For example, [16] characterized loci and genetically mapped the traits that influence anthocyanin pigmentation in potatoes. This study revealed that 21 pigmented out of 53 white cultivars shared a common bHLH allele, and this indicates the contribution of this allele, but this is also not adequate for completely pigmented cultivars. Studies on growth and physiological response, and breeding lack coordination and are therefore difficult to conclude. Yield data and the breeding of better yielding cultivars to aid in pigmented cultivar revitalization are severely lacking. This again shows the lack of data and the knowledge gap that needs to be filled by researchers around the world.

## 8. The Effect of Processing on Phytochemical Contents in Pigmented Potatoes

Potatoes including the pigmented cultivars need to be cooked (boiled, steamed, or fried) or processed into products such as crisps before consumption. As such, phytochemical compositions that are present before processing may change postprocessing and these may further change during absorption in the small intestines [87]. The antioxidant vitamin C, for example, is known to be heat-labile and, therefore, drastically deteriorates during cooking [87]. Therefore, vitamin C can perhaps be extracted more from fruits as they do not usually need to be cooked. In general, heat application is known to destabilize anthocyanins [88]. For example, frying reduced anthocyanins by 38–70% [45]. An earlier study of [46] had shown that anthocyanins were almost totally degraded by frying, while polyphenols remained stable, and antioxidants were significantly reduced. In contrast to these previous studies, [26] showed that anthocyanins increased in pigmented cultivars during cooking, although carotenoids decreased. In the previous authors’ study, boiling and microwaving significantly increased anthocyanins, while baking and frying did not significantly lead to an increase in the compounds. Boiling, on the other hand, increased antioxidant activity, while the other methods decreased these activities. However, the study of [51] showed that there was no change in phenolic acids in pigmented potatoes during cooking using the microwave or by boiling, while anthocyanins decreased by 16–29%. In another study, [53] showed that, and refractive window drying, drum drying, and freeze drying led to 23, 41, and 45% reductions in anthocyanin contents in pigmented cultivars, respectively. However, phenolic acids and antioxidant activity were not affected by drying in the previous study. The study of [47] reported that boiling and baking, respectively, led to 92 and 88% reductions in carotenoids. However, lutein and β-carotene were the most stable carotenoids in their study. The study of [54] also showed that snacks produced using pigmented potatoes were 20–30% richer in antioxidants and anthocyanins than the controls were, while polyphenols increased by up to 40%. Rytel et al. [63] reported that pre-drying and peeling pigmented potatoes led to significant losses in anthocyanins (75%), while polyphenols were significantly reduced (69%) by drying, blanching, and pre-drying during industrial processing. However, Furrer et al. [31] had earlier reported that the industrial processing of pigmented cultivars retained anthocyanins by 79–129%, while phenolics were retained by 49–85%. Although these studies are variable, carotenoids appear to be negatively affected by thermal processing, while polyphenols appear stable, and anthocyanins generally vary. As promising as these findings look, studies on the absorption and bioavailability of the compounds derived from pigmented potatoes need to be conducted to understand their real effect on human physiology.

## 9. Conclusions and Prospects

Pigmented potato cultivars are rare, and few studies have been conducted to document their characteristics. This review was able to reveal about 62 studies that have been conducted on pigmented cultivars around the world. Therefore, the dearth in the literature on physiological growth and yield, breeding, exhaustive secondary metabolite profiles, nutritional contents, absorption, and bioavailability studies and many other important studies is glaring. These studies would play an important role in revitalization efforts and the eventual commercialization of these important species. More studies need to be conducted to fill the missing knowledge gaps. The polyphenolic and antioxidant (anthocyanins and carotenoids) studies that have been conducted so far reveal the superior nature of these tuber crops in comparison with white-fleshed cultivars that are common throughout the world, and that have gained popularity in other applications that are too many to mention. Breeding studies need to concentrate on increasing the yield without compromising the phytochemical constituents. Yield has been shown to be one of the major factors limiting the commercialization of these species regardless of their promising health attributes. The antiproliferative studies that have been conducted so far look promising, but more could be done to reveal their full potential. The skins clearly contain higher phytochemical and biological constituents than the flesh parts and, therefore, consumers should be encouraged to explore the skins more. In addition, industrial applications of the skins, for example, in colorants, is clearly a viable option. What is clear from the work that has been conducted so far and the results is that pigmented potatoes are heavily underutilized, and they need to be revitalized and consequently commercialized. Their nutraceutical applications are glaring, and their functions potentially yield better results than the traditional, white-fleshed types. Some of the fears of the continued neglect of such species is extinction, and, if they go extinct, so do their potential food and nutrition security, as well as health/pharmaceutical benefits. Pigmented potatoes should not remain in the shadows, in high-end restaurants as gourmet foods, but should now trickle down to the wider populace for consumption and with the possibility to eventually replace the white-fleshed cultivars. Pigmented cultivars are healthier, and their consumption will potentially help to breed healthier nations through decreased lifestyle diseases and conditions. The proliferation of lifestyle diseases is costing governments billions in USD annually the world over and this problem needs to be nipped in the bud before it gets out of hand.

## Figures and Tables

**Table 1:** Distribution of pigmented potato cultivars in various regions of the world.

	Cultivar and Colour	Country	Ref.
1	Shepody, Desirée, 457-CON-1157, Corazón de buey, 302-UA-1634A, 304-UA-1135, (Boyo de chancho, Michuñeroja, Meca de gato, Cacho azu, Corazón azul, 239-UA-1388, Chona negra, and Bruja	Chiloe Island and Valdivia, Chile	[17]
2	NA	Louvain-La-Neuve, Belgium	[13]
3	NA	Louvain-La-Neuve, Belgium	[18]
4	704,429-Guincho Negra, 700,347-SS-2613, 702,535-Sipancachi, 701,997-Sullu, and 703,905-Huata Colorada	Louvain-La-Neuve, Belgium	[19]
5	Waicha—Reddish	Argentina	[20]
	Moradita—Purple		
6	Spunta, CN1—Pink	Sfax, Tunisia	[21]
7	No data	Global	[22]
8	Purple (PA97B29-2, PA97B29-4, PA97B29-5, and PA97B29-6)	Washington, DC, USA	[23]
	Red (PA97B29-3, PA97B35-1, PA97B35-2, PA97B36-3, PA97B37-2, PA97B37-3, PA97B37-7, PA97B39-2, and NDOP5847-1)		
9	Light Yellow (Adora, Divina, Fabula, Ilona, Morning gold, Provento, Satino, Yukon Gold, and POR00PG4-2	Washington, DC, USA	[24]
	Dark yellow (91E22, PA99P11-2, PA99P1-2, PA99P2-1, and POR00PG4-1)		
	Red and yellow (PO00PG9-1, PO00PG9-2, PO00PG9-3, PO00PG9-5, and PO00PG9-6)		
10	38 Cultivars	Washington, DC, USA	[25]
11	Purple (PA97B29-2, PA97B29-4, PA97B29-5, and PA97B29-6)	Washington, DC, USA	[26]
	Red (PA97B29-3, PA97B35-1, PA97B35-2, PA97B36-3, PA97B37-2, PA97B37-3, PA97B37-7, PA97B39-2, and NDOP5847-1)		
12	NA	Colorado, CO, USA	[27]
13	Hongyoung, Jayoung, and Atlantic	Dae-GwalLyeong, Korea	[15]
14	Hermanns Blaue, Highland Burgundy Red, Shetland Black, and Vitelotte	Barum, Germany 0.6–46 mg	[28]
15	NA	Sevilla, Spain	[29]
16	Congo—Purple	Bergen, Norway	[30]
17	Yellow (Innovator, Bintje, Challenger, Yukon, AR2009-10)	New Brunswick, Canada	[31]
	Purple (AR2, ADB)		
	White flesh, red skin (Norland)		
	Blue (Adirondack Blue)		
	Red (Adirondack Red, ADR),		
18	Purple (705,534, 703,640, 706,726, 704,733, 703,862, and 704,133)	Lima, Peru	[32]
	Red (702,556, 706,630, 700,234, 705,841, 703,752, 703,695, 705,820, 704,537, 705,946, 705,500, 702,464, and 703,782)		
	Yellow (706,884 and 704,481)		
19	Purple (Blue Congo, Blaue Veltlin, Blaue Schweden, Synkeä Sakari).	Finland	[33]
20	Purple (Blaue Elise, Blaue St. Galler, Blue Congo, Valfi, Violette, and Vitelotte) Red (Herbie 26, Highland B. Red, Rosalinde, and Rote Emma)	Prague, Czech Republic	[34]
21	Light purple—KM	Obihiro, Hokkaido, Japan	[35]
	Medium-dark purple—H92		
22	Yellow (Agria, Russet Burbank, Lady Balfour, and Mayan Gold)	Czech Republic	[36]
	Purple (Violette, Vitelotte, Violetta, Valfi, Blue Congo, Blaue St. Galler, Olivia, and Blaue Anneliese)		
	Red (Rosemarie, Rote Emmalie, Highland Burgundy Red, and Herbie 26)		
	Yellow with red spots (Mayan Queen)		
23	Salad Blue, Shetland Black, Blue Congo, Blaue St. Galler, Highland Burgundy Red, Violette, and Valfi, Vitelotte	Přerov nad Labem, Suchdol, Valečov and Stachy, Czech Republic	[37]
24	Hermanns Blaue, Vitelotte, Shetland Black, and Valfi	Braunschweig, Germany	[38]
25	Blue violet (Blaue Schweden, British Columbia Blue, Violettfleischige, 1.81.203–92N, Blaue Mauritius, Bleu, Blaue Utwill, Peru Purple, Mesabi Purple Smith’s Purple, UAC NEG 61, UAC CON 917, Weinberger Blaue, Mesabi Purple, Bells Purple, Magdeburger Blaue, Shetland Black, Caribe, Purple and White, Mrs. Moerles Purple Baker, Long Blue, Edzell Blue, Odenwa¨lder Blaue, Schwarze Ungarin, Arran Victory, UAC 1258, Purple Fiesta, Viola, and Blaue Zimmerli). Red (Sangre, Kefermarkter Zuchtstamm, and Red Cardinal).	Lenzen, Germany 0.01–1.57 g/kg	[39]
26	Review	Review	[40]
27	Purple—Hongyoung, Red—Jayoung	Korea	[41]
28	WP (Ranger Russet), YP (PORO3PG6–3), and PP (PORO4PG82–1)	Washington, USA	[42]
29	White (Russet Burbank), yellow (PORO3PG6-3), and purple-flesh (PORO4PG82-1)	Toppenish, Washington, USA	[43]
30	Red—Hongyoung	Korea	[44]
	Purple—Jayoung, clones Jje08-11,		
	DJ12X-5, and Jje08-43		
31	Purple (Salad Blue, Vitelotte, Valfi, Blue Congo)	Prerov nad Labem, Czech Republic	[45]
	Red (Rosalinde, Herbie 26, Highland Burgundy Red)		
32	Purple (Blaue Elise, Blaue St. Galler, Blue Congo, Valfi, and Vitelotte)	Přerov nad Labem, Czech Republic	[46]
	Red (Highland Burgundy Red, Herbie 26, Rosalinde, and Rote Emma)		
33	Yellow (Agria, Russet Burbank, Valy, Salome, Bohemia, Axa, Jelly, Ditta, Bionta, Kerˇkovsky’ rohlícˇek, Dali, and Mayan Gold)	Valečov, Czech Republic	[47]
	Red (Rosara, Rosemarie, Königspurpur, Highland Burgundy Red, Herbie 26, and Red Emmalie)		
	Purple (Valfi, Violeta, Blaue Anneliese, and Vitelotte)		
34	CO97226-2R/R, CO99364-3R/R, CO97215-2P/P, CO97216-3P/P, CO97227-2P/P, CO97222-1R/R, Purple Majesty, Mountain Rose, and All Blue), and yellow (Yukon Gold)	Colorado, USA	[48]
35	Blue Congo, Highland Burgundy Red, Salad Blue, Shetland Black, Valf, Vitelotte, Violette, Blaue St. Galler, Blaue Hindel Bank, Blaue Ludiano, Blaue Mauritius, Blaue Schweden, British Columbia Blue, Farbe Kartoffel, Hafija, and Salad Red	Czech Republic	[49]
36	HB Red, Rote Emma, Blaue St Galler, Valfi, Violette and Agria	Czech Republic	[50]
37	Vitelotte Noire and Highland Burgundy Red	Milan, Italy	[51]
38	Yellow (Agrie Dzeltenie, Prelma, Lenora, Brasla, Anuschka, Gundega, S04009-37)	Priekuli, Latvia	[52]
	Light yellow (S99108-8)		
	Purple (Fenton, Purple Fiesta, British Columbia		
	Blue, Purple Peru, and Blue Congo)		
39	Red (Red Rodeo) Yellow (Yukon Gold), and Purple Majesty (CO94165-3P/P)	Washington, USA	[53]
40	Red—Herbie 26	Poland	[54]
	Purple—Valfi, Blue Congo and Salad Blue		
41	Purple (Violettfleischige x Blue Marker-B, (Violettfleischige)-A1, (Blue Marker)-B, Purple, Violettfleischige x Blue Marker-D, (Violettfleischige)-A2, Vitelotte, and Blaue Ajanhuiri)	Groß Lüsewitz, Germany	[55]
	Yellow (Bangladesh, Desiree, Early Rose, and Shetland Blau I,)		
	Red (Ko¨nigspurpur, Rote Emmalie, and Rosemarie)		
42	Red (CO99256-2R, CO98012-5R, Colorado Rose, VC0967-2R/Y, CO97222-1R/R, and CO97226-2R/R)	Colorado, USA	[56]
	Purple (CO01399-10P/Y, AC99329-7PW/Y, and Purple Majesty)		
43	Purple (All Blue and CO94165-3P/P)	Texas, USA	[14]
	Red (NDC4069-4 and CO94183-1R/R)		
44	All Blue, NDC4069-4, Russian Blue, Purple Peruvian, COl11F2-1, COl12F1-1, COl12F1-2, CO141F2-1, CO142F2-1, RC2003-2	Colorado and Texas, USA	[57]
45	Purple (All Blue, Purple Peruvian, and RC2003-2)	Texas and Colorado, USA	[58]
	Red (NDC4069-4, CO94183-1R/R, and CO94183-1R/)		
46	NA	Oregon, USA	[59]
47	NA	Oregon, USA	[60]
48	NA	Oregon, USA	[61]
49	Blue—Valfi, Blaue Elise, Bore Volley, and Blue Congo	Přerov nad Labem, Czech Republic	[62]
50	Red—Rosemarie, Herbie 26, and Rote Emma	Wrocław, Poland	[63]
51	Red (Rosemary, Red Emmalie, and Red Cardinal)	Velestino, Greece	[64]
	Purple (Purple, Violetta, and Kefermarkter Blaue)		
52	Purple (Moradita)	Argentina	[65]
	Yellow (Waicha)		
	Red (Santa Marıa)		
53	Rio Grande, Mountain Rose, R Burbank, R Nugget, Yukon Gold, and Purple Majesty	Colorado, USA	[66]
54	Yellow (Satina and Tajfun, and Jelly)	Central Poland	[67]
55	Purple Majesty, Yukon Gold, Mountain Rose	Colorado, USA	[68]
56	Zheshu 13, 33, 75, 81, 132, 259, 6025	An’ji, Zhejiang Province, China	[69]
57	Red (Red Emmalie, Tornado, and Laura)	Širvintos district, Lithuania	[70]
	Dark purple (Violetta)		
	Dark-blue purple (Salad Blue)		
58	50 cultivars	Argentina	[71]
59	Salad Blue, Pink Fir Apple, and Highland Burgundy Red	Cape Town, South Africa	[11]
60	Salad Blue, Pink Fir Apple, and Highland Burgundy Red	Cape Town, South Africa	[72]
61	Agata, Cherie, Kennebec, Monalisa, Red Pontiac and Spirit	Barcelona, Spain	[10]
62	Purple Cloud No. 1, Red Cloud No. 1, Yunnan Potato 303, Yunnan Potato 603, S03-2677, S03-2685, S03-2796, S05-603, S06-277, and S06-1693	Chengdu, China 60–294 mg	[73]

NA: Not Available.

**Table 2:** Anthocyanins in pigmented potato cultivars.

	Anthocyanin	Ref.
Blue	Petunidin, Cyanidin, Delphinidin, Petunidin, Pelargonidin, Peonidin, Malvidin	[28,32,38,73]
Red	Pelargonidin, Cyanidin, Delphinidin, Petunidin, Peonidin, Malvidin	[28,32,38,73]
Purple	Peonidin, Petunidin, Delphinidin, Petunidin, Pelargonidin, Peonidin, Malvidin	[28,32,38,73]
Yellow	Malvidin, petunidin, Cyanidin, Delphinidin, Petunidin, Pelargonidin, Peonidin	[28,32,38,73]
Pink	Cyanidin, Delphinidin, Petunidin, Pelargonidin, Peonidin, Malvidin	[32,38,73]

## Data Availability

Not applicable.
